# Early Career Publication Trajectory of Male Infertility Fellows During and After Fellowship

**DOI:** 10.7759/cureus.36046

**Published:** 2023-03-12

**Authors:** Jesus Perez, Benjamin Plambeck, Christopher M Deibert

**Affiliations:** 1 Urology, University of Nebraska Medical Center, Omaha, USA; 2 Urology, University of Iowa Hospitals and Clinics, Iowa City, USA

**Keywords:** publication trends, male fertility, urology education, scholarly productivity, reproductive medicine

## Abstract

Background: Improving evidence-based medicine through research contribution is an important aspect of fellowship training. Prior studies have investigated the research activity of urology fellows during and after fellowship. The main objective of this study was to specifically explore the publication productivity of male infertility fellows both during fellowship and in the first 5 years afterwards.

Methods: The 19 fellowship program directors from the Society for the Study of Male Reproduction were asked to share a list of fellows from 2004 to 2019. Publications from 87 fellows at 12 programs were analyzed. PubMed® was utilized to search for peer-reviewed publications for each fellow during their fellowship and in the first 5 years afterwards. Each publication was classified by publication type (original research, review article, editorial comment, or case report), topic (fertility, testosterone, or other), and author type (sole, first, middle, or last author).

Results: Some 87 male infertility fellows were analyzed, and a total of 1608 peer-reviewed publications were identified. Some 662 total publications (41.2%) were written on the topic of fertility during fellowship and in the first 5 years afterwards. Some 554 (34.5%) publications were completed during fellowship, 178 (11.1%) in year 1, 164 (10.2%) in year 2, 220 (13.7%) in year 3, 269 (16.7%) in year 4, and 223 (13.9%) in year 5. The mean number of publications during fellowship was 6.37 (range 0-57). Means for years 1-5 after fellowship were 2.12, 1.95, 2.65, 3.36, and 2.97, respectively. After fellowship, 25.3% of the fellows did not publish again. A significant difference was detected between the group mean number of publications (analysis of variance, ANOVA - p = 0.0001) during fellowship and the 5 years afterwards. There was no significant difference between the group mean number of publications between the 1st and 5th years post-fellowship (ANOVA - p = 0.5919).

Conclusions: As anticipated, male infertility fellows were most productive during fellowship, with relatively stable research production thereafter. Thus, early career support and mentorship remain important to the future academic success of fellows. Future investigation of the relationship between male infertility fellow characteristics and the pursuit of an academic career is warranted.

## Introduction

Urology research productivity at the level of the medical student, resident, and fellow is a critical component of urology training and early career trajectory. While an extra research year in residency is significantly associated with increased career research productivity, any publication before residency is a significant predictor of an academic career [1]. In addition, the number of publications produced during urology residency is directly correlated with an increased pursuit of fellowship training and an academic career [2]. It is, thus, suggested that educational research be integrated into urology residency training with the goal of improving individual competence and consideration of an academic career [3]. However, little is known about the relationship between research productivity during urology fellowships and early career trajectory.

Improving evidence-based medicine through research contribution is an important component of fellowship training. Urology residents often seek fellowship training to gain focused knowledge of disease and surgical techniques, in addition to refining their research skills. Few have studied the productiveness and career paths of urology fellows after fellowship. One study investigated the publication productivity and early career trajectory of genitourinary reconstructive surgery (GURS) fellows [4]. However, the relationship between publication productivity and early career trajectory for male infertility fellows is poorly understood.

The main objective of this study was to explore the publication productivity of male infertility fellows both during fellowship and in the first 5 years afterwards. We hypothesized that the mean number of publications would be highest during fellowship and lowest during years 1-2 after fellowship, with increasing publication productivity thereafter. Secondly, we analyzed the quality of research performed according to parameters of publication type, topic, and authorship type. Lastly, we explored the relationship between academic productivity in fellowship and the pursuit of an academic career.

## Materials and methods

Fellowship program directors from the Society of Male Reproduction were contacted to provide a list of their fellow trainees from 2004 to 2019 and 12 male infertility fellowship programs provided their lists of fellows. We solely utilized PubMed® to search for publications produced by each fellow during fellowship and in the five years after fellowship. To exclude research work from residency, we correlated the year of the publication to the affiliated institution and principal investigator. Each publication was categorized by period (fellowship period and post-fellowship years 1-5), publication type (original research, review article, editorial comment, and case report), topic (fertility, testosterone, and other: erectile dysfunction, penile implant, Peyronie’s disease, other), and author type (first author, last author, sole author, and other: not first/last). 

We conducted a Google search to determine if the urologists had a history of working in an academic role. Urologists were categorized as academic urologists if they worked in an academic setting for any period post fellowship. Some urologists had concurrent roles in academic and private practice settings and were categorized as an academic role. This study portion was limited to their work history available within the first 20 google searches. Once we categorized academic vs. private practice urologists, we conducted statistical analysis to compare publication productivity.

Statistical analysis was performed using Microsoft Excel® and R® statistical software. Statistical tests employed include ANOVA, t-test two-sample assuming equal variances (one-tailed p-value), t-test two sample assuming unequal variances (one-tailed p-value), and Pearson’s correlation coefficient.

## Results

From the 12 programs that provided fellow names, a total of 1608 publications were produced by 87 fellows during fellowship and in the five years after fellowship. The mean number of publications produced during fellowship was 6.37 (range 0-57), and mean number of publications in the 5 years after fellowship was 2.11, 1.95, 2.65, 3.36, and 2.97, respectively. Some 29.7% of the total publications were review articles, 46.8% were original articles, 19.0% were editorial comments, and 4.5% were case reports. 

Some 34.4% of total publications were produced during fellowship. Subsequent productivity was 11.1% in post-year 1, 10.2% in post-year 2, 13.7% in post-year 3, 16.7% in post-year 4, and 13.9% in post-year 5. Greater publication productivity was demonstrated during fellowship vs. any other post-fellowship year (Table 1). There was no significant difference in publication productivity during the post-fellowship time periods (post-year 1-5) [F(4, 396) = 0.701, p = 0.592]. 11.5% of fellows did not publish during fellowship. Some 25.3% of fellows included in the study did not publish in the 5 years after fellowship.

**Table 1:** Publication productivity comparison between fellowship and the 5 years afterwards.

Comparison period	t-Test two sample (one-tailed p-value)
Fellowship vs. Post-year 1	t(172) = 3.46, p < 0.001
Fellowship vs. Post-year 2	t(172) = 4.28, p < 0.001
Fellowship vs. Post-year 3	t(172) = 3.40, p < 0.001
Fellowship vs. Post-year 4	t(171) = 2.66, p < 0.005
Fellowship vs. Post-year 5	t(168) = 2.81, p < 0.005

Some 45.3% of publications were classified as “other” for the topic category. “Other” included research topics not directly related to male infertility such as erectile dysfunction and penile implants. 41.2% of publications were classified as “fertility” and 13.5% of publications were classified as “testosterone therapy.” There was no significant difference in the mean number of “fertility,” “testosterone related,” and “other” publications per fellow when comparing the fellowship period and post-fellowship years (ANOVA values reported in order) [F(5, 340) = 1.50, p = 0.19], [F(5, 340) = 1.98, p = 0.080], and [F(5, 340) = 1.74, p = 0.125].

**Figure 1:**
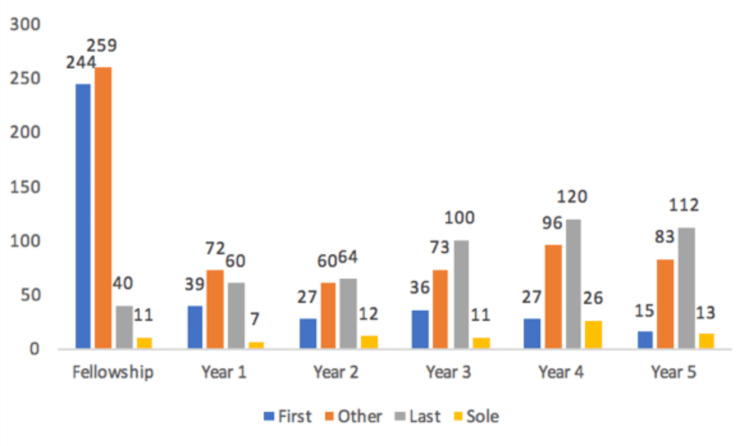
Productivity categorized by author type: first author, other: not first/last, last author, sole author.

First authorship was highest in fellowship, making up 44% of the publications during fellowship (Figure 1). The proportion of first authorship publications trended downwards in the 5 years following fellowship with proportions of 21.9%, 16.5%, 16.4%, 10.0%, and 6.7%, respectively [r(4)= -0.897, p = 0.015]. “Other” authorship did not demonstrate a significant relationship [r(4) = -0.735, p = 0.096]. Last authorship was 7.2% of the publications during fellowship and trended upwards with proportions of 33.7%, 39.0%, 45.5%, 44.6% and 50.2%, respectively [r(4)= 0.875, p = 0.023]. Sole authorship did not demonstrate a significant linear trend during fellowship and in the years after [r(4) = 0.679, p = 0.137].

The mean number of publications during fellowship for those that pursued an academic career (n = 51) was 7.35 and the mean number of publications produced by the private practice group (n = 36) was 4.97 [t(82) = 1.32, p = 0.099]. When comparing the mean number of publications produced by the academic group versus the non-academic group in each of the 5 years following fellowship, those in an academic setting published at a statistically higher rate in all post-graduate years (Table 2).

**Table 2:** Mean publication productivity comparing male infertility fellowship-trained urologists in an academic vs. non-academic role.

Time period	Academic	Non-academic	t-test (two-sample: unequal variances)
Fellowship	7.35	4.97	t(84) = 1.29, p = 0.099
Post-year 1	3.20	0.42	t(51) = 2.12, p = 0.019
Post-year 2	2.75	0.66	t(61) = 3.31, p < 0.001
Post-year 3	3.53	1.11	t(82) = 2.37, p = 0.010
Post-year 4	4.52	1.19	t(72) = 2.61, p < 0.01
Post-year 5	4.24	0.44	t(50) = 2.65, p < 0.01

## Discussion

As expected, our study showed fellows were most productive during fellowship (six publications), with relatively stable productivity in the 5 years thereafter. In addition, during fellowship a significant proportion of publications were written as first author, with a natural transition to principal investigator in early career. This was demonstrated by the significant upward trend in last author publications 5 years post-fellowship, while first authorship reciprocally decreased. Regarding research topic, the distribution of “fertility,” “testosterone,” and “other” publications did not differ significantly. Furthermore, we found that about 60% of the male infertility fellows included in this study have practiced or are currently in an academic career since completing their fellowship. Compared to the non-academic group, fellows pursuing an academic career published more papers (mean of 7.35 vs. 4.97) during their fellowships, though this difference was not statistically significant. On the other hand, publication productivity was significantly higher in the years after fellowship for the academic group, which is to be expected from an academic career.

Another study also investigated the publication productivity and early career trajectory of fellowship-trained urologists. These researchers found that genitourinary reconstructive surgeons (GURS) fellows published about four papers during fellowship. They also reported that GURS fellow publication productivity dwindled during early career [4]. In contrast, our findings showed that, although male infertility fellowship-trained urologists published fewer articles after fellowship, they maintained a consistent mean of two to three publications per year and, thus, productivity was sustained in their early career. However, among fellows that did not pursue an academic career, publication productivity was only about one publication per year.

Fellowship is a fertile training ground for learning research and clinical skills that translate into careers for many urologists, whether pursuing academics or private practice. This study is the first to explore the research productivity of male infertility fellows and how it relates to career trajectory and our findings help solidify the importance of early career support within fellowship for the academic success of male infertility fellows. In addition, although the difference was not statistically significant, male infertility fellows who pursued academic careers published more papers during their fellowships than those in the non-academic group. This could have implications toward the characteristics of male infertility fellows who choose to pursue academic careers, as well as help guide future mentorship and career planning for fellows.

The number of publications was our study's primary metric for academic productivity. A previous study using the H-index tool as a metric for analyzing research productivity of any fellowship-trained urologists found it was 3.44 times more likely to have an H index greater than the median [5]. Another study showed no difference between the H index of fellowship and non-fellowship-trained academic urologists [6]. Future studies should be conducted to examine the quality of research productivity in fellowship-trained male infertility specialists in an academic vs. non-academic practice. We did not include H-index in the current study.

Our study is not without limitations. We reviewed 1608 publications of 87 fellows from 12 of 19 programs. Thus, not all programs participated, nor were prior fellows listed on their websites. In addition, PubMed® was the only source utilized for our publication search. As a result, articles published in journals lacking PubMed® indexing were not included. Furthermore, name misspellings at the journal level could have led to inadvertent exclusion of publications as well.

## Conclusions

Research productivity of fellows in male infertility is highest during fellowship, with reasonable consistency in subsequent years. Thus, the early career support and mentorship that arises from fellowship training continues to be a vital component of future academic success for male infertility fellowship-trained urologists. Future work should be done to further elucidate the relationship between male infertility fellow characteristics and the pursuit of an academic career.
